# Identification of *SUPT3H* as a novel 8q24/*MYC* partner in blastic plasmacytoid dendritic cell neoplasm with t(6;8)(p21;q24) translocation

**DOI:** 10.1038/bcj.2015.26

**Published:** 2015-04-10

**Authors:** Y Nakamura, H Kayano, E Kakegawa, H Miyazaki, T Nagai, Y Uchida, Y Ito, N Wakimoto, S Mori, M Bessho

**Affiliations:** 1 Department of Hematology, Saitama Medical University, Saitama, Japan; 2 Department of Pathology, Saitama Medical University, Saitama, Japan

Blastic plasmacytoid dendritic cell neoplasm (BPDCN) (previously referred as blastic natural killer cell leukemia/lymphoma or agranular CD4+/CD56+ hematodermic neoplasm) is a rare hematologic malignancy derived from the precursors of plasmacytoid dendritic cells and is now classified as a rare subtype of acute myeloid leukemia according to the 2008 WHO classification.^1^ The disease is a clinically aggressive tumor with a high frequency of cutaneous and bone marrow (BM) involvement. Tumor cells express CD4, CD43, CD45RA and CD56, as well as the plasmacytoid dentritic cell-associated antigens CD123, BDCA-2/CD303, TCL1 and CTLA1.

The pathogenesis of BPDCN is largely unknown. Previous genetic study reported that complex chromosomal aberrations, such as deletion of 5q, 12p, 13q, 6q, 15q or 9, were observed in most cases with BPDCN.^2^ A reciprocal chromosomal translocation, t(6;8)(p21;q24), was reported in four cases with BM infiltration^2, 3, 4, 5^ and it appears to be a recurrent cytogenetic abnormality in BPDCN. Here, we report the identification of *SUPT3H* at 6p21 as a novel non-immunoglobulin (*Ig*) partner gene of 8q24 translocation in a patient with BPDCN presenting t(6;8)(p21;q24).

The patient was an 81-year-old man who visited our hospital because of cervical lymphadenopathy and skin tumor. He was found to have pancytopenia with hemoglobin level of 7.9 g/dl, a white blood cell count of 3.2 × 10^9^ /l and a platelet count of 46 × 10^9^ /l. Serum LDH level was highly elevated to 12780 IU/l. Lymph node (LN) biopsy presented diffuse infiltration of atypical cells. Results of flow cytometry and immunohistochemistry analysis revealed that tumor cells were positive for CD4, CD7 and CD56, but negative for other myeloid or lymphoid markers including CD3, CD8, CD20, CD21, TIA1, granzyme B or myeloperoxidase. On the basis of immunophenotypic findings, he was diagnosed as BPDCN. BM was highly infiltrated with tumor cells. He was admitted for chemotherapy, but died of respiratory failure after the development of intracranial hemorrhage.

The results of conventional chromosome analysis were as follows; 47,X,-Y t(6;8)(p21;q24),+add(7)(p11.2),+der(8)t(6;8),+20 [17/20], 46,XY [3/20] among LN cells; 48,X,-Y,t(6;8)(p21;q24),+add(7)(p11.2),+der(8)t(6;8),+20 [5/13], 49, idem,+mar1 [2/13], 49,idem,der(8)t(6;8),?t(9;15)(p22;q15),+mar1 [2/13], 46, XY [3/13] among BM cells (Figure 1a).

We postured the possible involvement of *MYC* in t(6;8)(p21;q24) translocation and performed fluorescence *in situ* hybridization analysis using the 8q24 probe-LSI MYC Dual Color, Break Apart Rearrangement Probe, consisting of the SpectrumOrange-labeled 5' LSI MYC probe, which begins at 119 kb upstream of the 5′ end of MYC and extends 266 kb toward the centromere, and the SpecrumGreen-labeled 3′ LSI MYC probe, which starts approximately at 3′ of MYC and extends 407 kb toward the telomere (Abbott Japan, Tokyo, Japan). The result showed the splitting of hybridization signals on translocated chromosomes, indicating that the 8q24 chromosomal break occurred within or around *MYC* (Figure 1b). However, DNA rearrangement within *MYC* gene was not detected by long-distance inverse PCR method.^6^ Immunohistochemistry of biopsied LN, using anti-c-Myc (N-term) rabbit monoclonal antibody (Epitomics, CA, USA), presented high expression of MYC protein in nucleus of the tumor cells (Figure 1c).

To determine 8q24 partner gene, we searched the chimeric transcript using the 3' rapid amplification cDNA end method, as frequent rearrangements within *PVT1* locus locating 57 kb 3′ of *MYC* were recently reported in multiple myeloma with 8q24 abnormality.^7^ Total RNA was extracted from the patient's BM and LN cells and reverse-transcribed using a QT primer (5′-TGAGCAGAGTGACGAGGAGGACTCGAGCTCAAGCTTTTTTTTTTTTTTTTT-3′). cDNA was amplified with a specific primer, PVT1-F (5′-CTGTGACCTGTGGAGACACGG-3′), which corresponds to chromosome 8 sequence (nucleotides 128847191 to 128847211 of the reported genomic sequence: NCBI Reference Sequence NC_018919.2) and universal primer Q0 (5′-CCAGTGAGCGAGTGACG-3′). An aliquot of this reaction product was further amplified using PVT1-F and semi-nested universal primer Q1 (5′-GAGGACTCGAGCTCAAGC-3′).

Abnormal products were amplified from the patient's LN and BM samples. The result of sequence of PCR products revealed that chromosome 8 sequence (previously referred as *PVT1* exon 1) was fused to exon 3 of *SUPT3H* (suppressor of Ty 3 homolog (*Saccharomyces cerevisiae*)), which had been assigned to chromosome 6p21,^8^ indicating the 6p21 chromosomal breakpoint was located within *SUPT3H* gene (Figure 2a).

The formation of the chimeric transcript was ascertained by RT-PCR analysis using PVT1-F and a primer from *SUPT3H* exon 7 (5′-CCATACTGCTGCAGTCCAACC-3′) (Figures 2b and c). Sequencing of the PCR product revealed that the *SUPT3H* part corresponded to type 1 transcript of the gene. Reciprocal transcript was not detected by RT-PCR. *SUPT3H* was also expressed from untranslocated allele in the patient's tumor cells as shown by RT-PCR using *SUPT3H* exon 2 (5′-AGCTAGTCCAATGTCTACTGC-3′) and exon 7 primers (Figures 2b and c).

Translation initiation site of *SUPT3H* was located at 5' side from the chromosome 6 breakpoint and an open reading frame, which started from chromosome 8 and fused in frame to *SUPT3H*, was generated in the chimeric transcript. The predicted product was truncated by 17 amino acids at N-terminus when compared with the wild-type SUPT3H protein.

SUPT3H is a TATA-binding protein-associated factors (TAF)-associated protein that composes of the human histone acetyl transferase STAGA complex (SPT3-TAF9-GCN5-acetylase)^9, 10^ and is ubiquitously expressed in all tissues. Recently, a possible association between variation (interstitial duplication) in this gene and 46, XY gonadal dysgenesis was suggested,^11^ but, to our knowledge, its involvement in chromosomal translocation has not been reported so far.

The 8q24/*MYC* chromosomal translocations with *Ig* gene loci are the major genetic aberrations in Burkitt's lymphoma. Recently, occasional occurrence of 8q24 rearrangements with non-*Ig* partners in B-cell lymphoma and multiple myeloma was reported.^6, 7^ In myeloid tumor, five cases with acute myeloid leukemia exhibiting t(8;14)(q24;q32) chromosomal translocation were reported, previously.^11, 12, 13, 14, 15^ In one of them, fluorescence *in situ* hybridization analysis revealed an atypical 8q24 rearrangement without involvement of *Ig* heavy chain gene at 14q32 or *MYC* overexpression, suggesting that the rare t(8;14) occurring in AML may differ molecularly from that observed in B-cell tumor.^14^ To our knowledge, our case is the first instance of myeloid malignancy associated with 8q24 chromosomal translocation affecting *MYC*.

In our case, it was not determined whether or not the truncated SUPTH3H protein from the chimeric transcript was expressed in tumor cells or the structural alteration of the protein contributed to the cellular transformation. Thus, significance of the formation *PVT1-SUPT3H* chimeric gene remains unknown and the overexpression of *MYC* by ectopic promoter on chromosome 6 may be essential to tumor genesis. However, our results indicate that 8q24 translocation may exert as a molecular pathogenesis in non-lymphoid hematologic neoplasms. Further investigation is needed to clarify the role of the chromosomal aberration in pathogenesis of BPDCN.

## Figures and Tables

**Figure 1:**
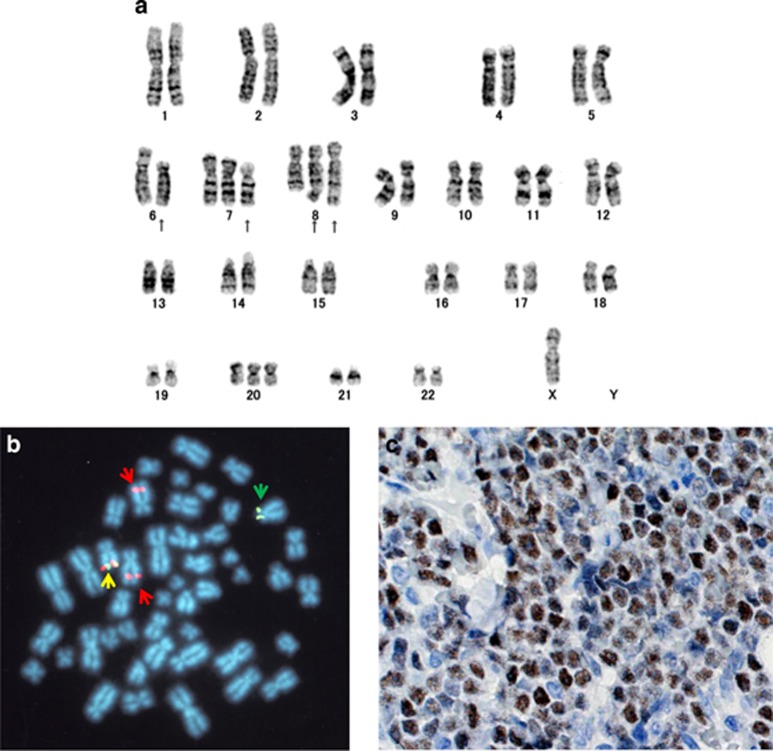
(**a**) G-banded karyogram of the patient's BM cells. Arrows indicate the aberrant chromosomes. (**b**) Fluorescence *in situ* hybridization analysis of BM cells using Spectrum Orange-labeled 5' LSI MYC and Spectrum Green-labeled 3' LSI MYC probes. Normal chromosome 8 is shown by fusion of the two probes (a yellow arrow). Derivative 6 and 8 chromosomes are indicated by green and red arrows. (**c**) Immunohistochemistry of biopsied LN using anti-MYC antibody. MYC was highly expressed in nucleus of tumor cells.

**Figure 2:**
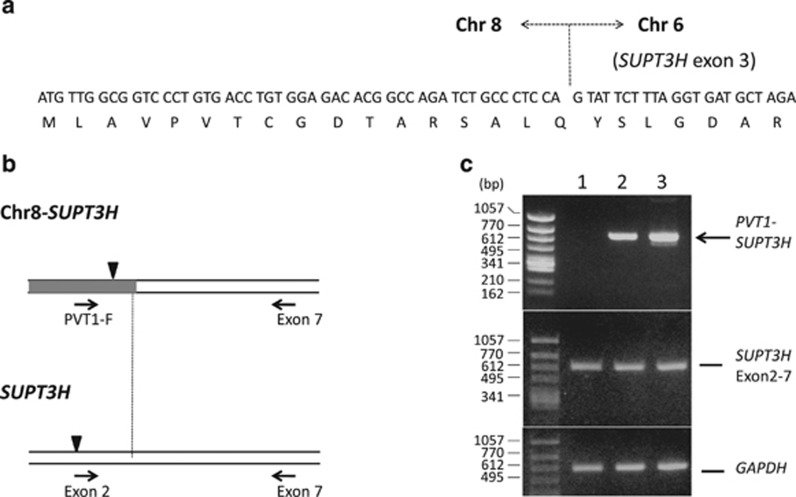
(**a**) Partial nucleotide and deduced amino acid sequence around the junction of the fusion transcript. (**b**) Schematic representation of the fusion transcript. Horizontal arrows indicate primers used in RT-PCR. Triangles indicate translation initiation sites. (**c**) RT-PCR analysis, detecting the fusion and the normal *SUPT3H* transcripts. Lanes 1, 2 and 3 indicate peripheral mononuclear cells from a normal individual, the patient's LN and BM cells, respectively.
